# 5-Amino-1-methyl-1*H*-benzimidazole

**DOI:** 10.1107/S1600536809025550

**Published:** 2009-07-08

**Authors:** Jan Lokaj, Viktor Kettmann, Viktor Milata, Tomáš Solčan

**Affiliations:** a Faculty of Food and Chemical Technology, Slovak Technical University, Radlinskeho 9, SK-81237 Bratislava, Slovak Republic; b Faculty of Pharmacy, Comenius University, Odbojarov 10, SK-83232 Bratislava, Slovak Republic

## Abstract

The structure of the title compound, C_8_H_9_N_3_, a potential anti­tumour drug, was determined in order to give more insight into its structure–function relationships. The benzimidazole core of the mol­ecule was found to be exactly planar, while the substituents are displaced slightly from the mol­ecular plane [C—C—N—C and C—C—C—N torsion angles of 0.8 (3) and 179.0 (1)° for the methyl and amino groups, respectively]. The bond lengths are analysed in detail and compared with those of the parent unsubstituted analogues. The results show that the lone-pair electrons on the amino N atom are involved in conjugation with the adjacent π system and hence affect the charge distribution in the heterocycle. Two inter­molecular N—H⋯N and C—H⋯N hydrogen bonds have been identified.

## Related literature

For the synthesis, see: Milata *et al.* (1989 ▶). For bond-order–bond-length curves, see: Burke-Laing & Laing (1976 ▶). For the biological activity of benzimidazole derivatives, see: Kettmann *et al.* (2004 ▶); Le *et al.* (2004 ▶); Nguyen *et al.* (2004 ▶); Statkova-Abeghe *et al.* (2005 ▶). For a description of the Cambridge Structural Database, see: Allen (2002 ▶).
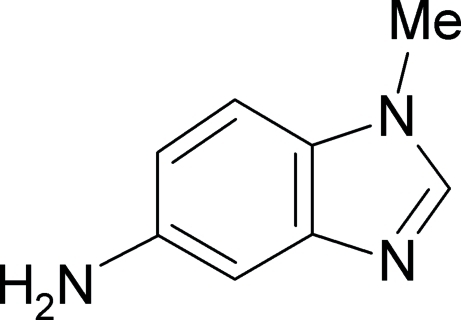


## Experimental

### 

#### Crystal data

C_8_H_9_N_3_
*M* *_r_* = 147.18
Monoclinic, 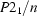

*a* = 5.9128 (2) Å
*b* = 8.8215 (3) Å
*c* = 14.8418 (6) Å
β = 100.129 (3)°
*V* = 762.08 (5) Å^3^
*Z* = 4
Mo *K*α radiation
μ = 0.08 mm^−1^
*T* = 296 K
0.52 × 0.20 × 0.10 mm

#### Data collection

Oxford Diffraction Gemini R CCD diffractometer
Absorption correction: analytical [*CrysAlis RED* (Oxford Diffraction, 2009 ▶) based on Clark & Reid (1995 ▶)] *T*_min_ = 0.944, *T*_max_ = 0.966
18508 measured reflections
1832 independent reflections
1114 reflections with *I* > 2σ(*I*)
*R*_int_ = 0.039

#### Refinement

*R*[*F* ^2^ > 2σ(*F* ^2^)] = 0.046
*wR*(*F* ^2^) = 0.141
*S* = 1.02
1832 reflections
101 parameters
H-atom parameters constrained
Δρ_max_ = 0.21 e Å^−3^
Δρ_min_ = −0.21 e Å^−3^

### 

Data collection: *CrysAlis CCD* (Oxford Diffraction, 2009 ▶); cell refinement: *CrysAlis RED* (Oxford Diffraction, 2009 ▶); data reduction: *CrysAlis RED*; program(s) used to solve structure: *SHELXS97* (Sheldrick, 2008 ▶); program(s) used to refine structure: *SHELXL97* (Sheldrick, 2008 ▶); molecular graphics: *ORTEP-3* (Farrugia, 1997 ▶); software used to prepare material for publication: *enCIFer* (Allen *et al.*, 2004 ▶).

## Supplementary Material

Crystal structure: contains datablocks global, I. DOI: 10.1107/S1600536809025550/ez2173sup1.cif

Structure factors: contains datablocks I. DOI: 10.1107/S1600536809025550/ez2173Isup2.hkl

Additional supplementary materials:  crystallographic information; 3D view; checkCIF report

## Figures and Tables

**Table 1:** Hydrogen-bond geometry (Å, °)

*D*—H⋯*A*	*D*—H	H⋯*A*	*D*⋯*A*	*D*—H⋯*A*
N5—H5*A*⋯N3^i^	0.86	2.47	3.1447 (19)	136
C2—H2⋯N5^ii^	0.93	2.58	3.503 (2)	171

Symmetry codes: (i) 
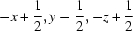
; (ii) 
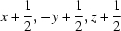
.

**Fig. 1.**
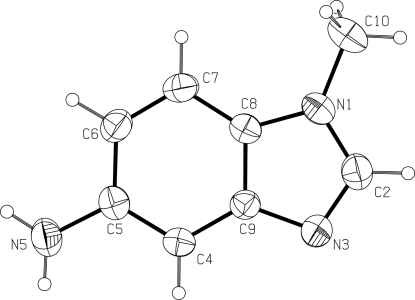
Perspective view of (I), with the atom numbering scheme. Thermal ellipsoids are drawn at the 50% probability level.

## supplementary crystallographic information

### Comment

Benzimidazole derivatives are known to possess a variety of biological properties (Le *et al.*, 2004), the anti-cancer activity being one of the most important (Nguyen *et al.*, 2004). Previously, it was shown that (*a*) introduction of a small substituent to the benzo-ring of 1*H*-benzimidazoles has a profound effect on the cytotoxic activity (Statkova-Abeghe *et al.*, 2005) and (2) the activity is related to intercalative interaction of the drug molecule with the nuclear DNA or the DNA-topoisomerase binary complex (Kettmann *et al.*, 2004). It is, however, unclear whether the influence of the substituents reflects their effect on the charge distribution of the heterocycle (and hence the intercalative energy) or results from interaction of the substituents with additional DNA or enzyme functionalities. Consequently, we prepared a series of substituted 1-methylbenzimidazoles and determined and compared their molecular and electronic structures by using theoretical and experimental techniques. In this communication we report the crystal structure of the 5-amino derivative, (I).

As expected, the ring system of the molecule is planar (Fig.1) to within experimental error and the substituents are slightly displaced to the same side of the plane, as indicated by torsion angles of 0.8 (3)° (C7-C8-N1-C10) and 179.0 (1)° (C9-C4-C5-N5) for the methyl and amino groups, respectively.

As mentioned above, the main purpose of this work was to compare precise molecular dimensions in the present derivative, (I), with those of the unsubstituted 1-methylbenzimidazole. As the latter compound has no entry in the Cambridge Structural Database (CSD, version 5.30 of November 2008; Allen, 2002), the CSD was searched for compounds possessing the benzimidazole core and just one substituent with the methylene group in the α-position; 42 such compounds [hereafter (II)] were found. The comparison has shown that the corresponding bond lengths in the benzimidazole heterocycle in (I) and in the molecules of (II) are significantly different. More specifically, the corresponding bond lengths are [the first number concerns compound (I), second number represents an average through 42 compounds (II)]: C4—C5: 1.382 (2), 1.355 (3); C5—C6: 1.410 (2), 1.387 (3);  C6—C7: 1.370 (2), 1.387 (3); C7—C8: 1.391 (2), 1.370 (3);  C8—C9: 1.393 (2), 1.397 (2);  C9—C4: 1.389 (2), 1.403 (3) Å. This, along with the partial double-bond character of C5–N5 (according to the bond-order - bond-length curves proposed by Burke-Laing & Laing, 1976) indicates that the amino group is conjugated with the benzimidazole ring. This further implies that for the present derivative the intercalative energy makes an important contribution to the overall drug-DNA binding energy and hence the enhanced cytotoxic activity of (I) relative to (II) (Kettmann *et al.*, 2004). These results will serve as a basis for subsequent molecular-modelling studies of the DNA-enzyme-ligand interactions.

The crystal packing is dominated by two intermolecular N–H···N and C–H···N hydrogen bonds (Table 1). It is notable that the amino N5 atom accepts a (weak) hydrogen bond but only one of the two N–H donors is involved in hydrogen bonding.

### Experimental

As described in detail previously (Milata *et al.*, 1989), the title compound, (I), was synthesized by starting from 2,4-dinitrochlorobenzene *via* nucleophilic substitution of the chlorine with methylamine, followed by partial Zinnin reduction of the *ortho*-nitro group and subsequent cyclization to obtain 1-methyl-5-nitrobenzimidazole which after reduction gives the target compound (m.p. 430–432 K).

### Refinement

H atoms were visible in difference maps and were subsequently treated as riding atoms with distances C—H = 0.93 Å (CH_arom_), 0.96 Å (CH_3_) and N—H = 0.86 Å; *U*_iso_ of the H atoms were set to 1.2 (1.5 for the methyl H atoms) times *U*_eq_ of the parent atom.

### Crystal data

**Table:**

C_8_H_9_N_3_	*F*(000) = 312
*M**_r_* = 147.18	*D*_x_ = 1.283 Mg m^−^^3^
Monoclinic, *P*2_1_/*n*	Melting point: 431 K
Hall symbol: -P 2yn	Mo *K*α radiation, λ = 0.71073 Å
*a* = 5.9128 (2) Å	Cell parameters from 7029 reflections
*b* = 8.8215 (3) Å	θ = 3.5–29.5°
*c* = 14.8418 (6) Å	µ = 0.08 mm^−^^1^
β = 100.129 (3)°	*T* = 296 K
*V* = 762.08 (5) Å^3^	Needle, orange
*Z* = 4	0.52 × 0.20 × 0.10 mm

### Data collection

**Table:**

Oxford Diffraction Gemini R CCD diffractometer	1832 independent reflections
Radiation source: fine-focus sealed tube	1114 reflections with *I* > 2σ(*I*)
graphite	*R*_int_ = 0.039
Detector resolution: 10.434 pixels mm^-1^	θ_max_ = 28.0°, θ_min_ = 3.5°
ω scans	*h* = −7→7
Absorption correction: analytical [*CrysAlis RED* (Oxford Diffraction, 2009) based on Clark & Reid (1995)]	*k* = −11→11
*T*_min_ = 0.944, *T*_max_ = 0.966	*l* = −19→19
18508 measured reflections	

### Refinement

**Table:**

Refinement on *F*^2^	Primary atom site location: structure-invariant direct methods
Least-squares matrix: full	Secondary atom site location: difference Fourier map
*R*[*F*^2^ > 2σ(*F*^2^)] = 0.046	Hydrogen site location: inferred from neighbouring sites
*wR*(*F*^2^) = 0.141	H-atom parameters constrained
*S* = 1.02	*w* = 1/[σ^2^(*F*_o_^2^) + (0.086*P*)^2^] where *P* = (*F*_o_^2^ + 2*F*_c_^2^)/3
1832 reflections	(Δ/σ)_max_ = 0.001
101 parameters	Δρ_max_ = 0.21 e Å^−^^3^
0 restraints	Δρ_min_ = −0.21 e Å^−^^3^

### Special details

**Table:**

Experimental. Analytical numeric absorption correction using a multifaceted crystal model based on expressions derived by Clark & Reid (1995).
Geometry. All e.s.d.'s (except the e.s.d. in the dihedral angle between two l.s. planes) are estimated using the full covariance matrix. The cell e.s.d.'s are taken into account individually in the estimation of e.s.d.'s in distances, angles and torsion angles; correlations between e.s.d.'s in cell parameters are only used when they are defined by crystal symmetry. An approximate (isotropic) treatment of cell e.s.d.'s is used for estimating e.s.d.'s involving l.s. planes.Least-squares planes (*x*,*y*,*z* in crystal coordinates) and deviations from them (* indicates atom used to define plane)2.7140 (0.0026) *x* - 6.5500 (0.0019) *y* + 5.9298 (0.0054) *z* = 1.2109 (0.0022)* -0.0006 (0.0010) N1 * -0.0119 (0.0012) C2 * -0.0077 (0.0011) N3 * 0.0063 (0.0010) C4 * -0.0054 (0.0011) C5 * -0.0129 (0.0011) C6 * 0.0007 (0.0011) C7 * 0.0188 (0.0012) C8 * 0.0127 (0.0012) C9 - 0.0337 (0.0017) N5 - 0.0311 (0.0023) C10Rms deviation of fitted atoms = 0.0103
Refinement. Refinement of *F*^2^ against ALL reflections. The weighted *R*-factor *wR* and goodness of fit *S* are based on *F*^2^, conventional *R*-factors *R* are based on *F*, with *F* set to zero for negative *F*^2^. The threshold expression of *F*^2^ > σ(*F*^2^) is used only for calculating *R*-factors(gt) *etc*. and is not relevant to the choice of reflections for refinement. *R*-factors based on *F*^2^ are statistically about twice as large as those based on *F*, and *R*- factors based on ALL data will be even larger.

### Fractional atomic coordinates and isotropic or equivalent isotropic displacement parameters (Å^2^)

**Table:**

	*x*	*y*	*z*	*U*_iso_*/*U*_eq_	
N1	−0.0430 (2)	0.25393 (13)	0.50429 (8)	0.0589 (4)	
C2	0.1513 (3)	0.32925 (18)	0.49663 (12)	0.0677 (5)	
H2	0.2202	0.3987	0.5401	0.081*	
N3	0.2352 (2)	0.29646 (15)	0.42273 (9)	0.0675 (4)	
C4	0.0831 (2)	0.11583 (16)	0.29522 (9)	0.0546 (4)	
H4	0.1996	0.1333	0.2618	0.066*	
C5	−0.0929 (2)	0.01590 (15)	0.26336 (10)	0.0561 (4)	
N5	−0.1043 (2)	−0.05967 (15)	0.18037 (9)	0.0746 (4)	
H5A	−0.0002	−0.0447	0.1475	0.090*	
H5B	−0.2155	−0.1214	0.1619	0.090*	
C6	−0.2678 (2)	−0.00852 (17)	0.31522 (11)	0.0646 (5)	
H6	−0.3861	−0.0754	0.2930	0.077*	
C7	−0.2698 (2)	0.06273 (17)	0.39712 (12)	0.0634 (4)	
H7	−0.3862	0.0452	0.4306	0.076*	
C8	−0.0905 (2)	0.16230 (15)	0.42807 (9)	0.0509 (4)	
C9	0.0833 (2)	0.18977 (15)	0.37784 (10)	0.0512 (4)	
C10	−0.1765 (3)	0.2690 (2)	0.57695 (12)	0.0796 (5)	
H10A	−0.0975	0.3348	0.6237	0.119*	
H10B	−0.1962	0.1711	0.6027	0.119*	
H10C	−0.3243	0.3112	0.5525	0.119*	

### Atomic displacement parameters (Å^2^)

**Table:**

	*U*^11^	*U*^22^	*U*^33^	*U*^12^	*U*^13^	*U*^23^
N1	0.0648 (8)	0.0597 (7)	0.0537 (8)	0.0113 (6)	0.0147 (6)	0.0011 (6)
C2	0.0660 (10)	0.0668 (9)	0.0681 (11)	0.0043 (8)	0.0053 (8)	−0.0118 (8)
N3	0.0594 (7)	0.0701 (8)	0.0745 (9)	−0.0073 (6)	0.0156 (6)	−0.0147 (7)
C4	0.0524 (7)	0.0571 (8)	0.0564 (9)	0.0039 (6)	0.0155 (6)	0.0016 (7)
C5	0.0588 (8)	0.0517 (8)	0.0554 (9)	0.0108 (6)	0.0037 (6)	−0.0003 (6)
N5	0.0777 (9)	0.0769 (9)	0.0664 (9)	0.0007 (7)	0.0047 (7)	−0.0173 (7)
C6	0.0566 (8)	0.0562 (9)	0.0806 (12)	−0.0067 (7)	0.0115 (8)	−0.0060 (8)
C7	0.0562 (8)	0.0606 (9)	0.0779 (11)	−0.0014 (7)	0.0243 (7)	0.0048 (8)
C8	0.0541 (8)	0.0483 (7)	0.0513 (8)	0.0090 (6)	0.0116 (6)	0.0050 (6)
C9	0.0465 (7)	0.0493 (7)	0.0580 (8)	0.0032 (6)	0.0099 (6)	−0.0005 (6)
C10	0.0964 (12)	0.0869 (12)	0.0616 (10)	0.0198 (9)	0.0307 (9)	0.0024 (9)

### Geometric parameters (Å, °)

**Table:**

N1—C2	1.350 (2)	N5—H5A	0.8600
N1—C8	1.3785 (18)	N5—H5B	0.8600
N1—C10	1.450 (2)	C6—C7	1.370 (2)
C2—N3	1.313 (2)	C6—H6	0.9300
C2—H2	0.9300	C7—C8	1.391 (2)
N3—C9	1.3879 (19)	C7—H7	0.9300
C4—C5	1.382 (2)	C8—C9	1.3926 (19)
C4—C9	1.3888 (19)	C10—H10A	0.9600
C4—H4	0.9300	C10—H10B	0.9600
C5—N5	1.3917 (19)	C10—H10C	0.9600
C5—C6	1.410 (2)		
			
C2—N1—C8	105.84 (12)	C7—C6—H6	118.8
C2—N1—C10	126.90 (14)	C5—C6—H6	118.8
C8—N1—C10	127.25 (14)	C6—C7—C8	117.22 (13)
N3—C2—N1	114.48 (14)	C6—C7—H7	121.4
N3—C2—H2	122.8	C8—C7—H7	121.4
N1—C2—H2	122.8	N1—C8—C7	132.41 (13)
C2—N3—C9	104.04 (12)	N1—C8—C9	105.93 (12)
C5—C4—C9	119.04 (13)	C7—C8—C9	121.62 (13)
C5—C4—H4	120.5	N3—C9—C4	129.96 (12)
C9—C4—H4	120.5	N3—C9—C8	109.71 (12)
C4—C5—N5	121.66 (14)	C4—C9—C8	120.32 (13)
C4—C5—C6	119.40 (14)	N1—C10—H10A	109.5
N5—C5—C6	118.93 (14)	N1—C10—H10B	109.5
C5—N5—H5A	120.0	H10A—C10—H10B	109.5
C5—N5—H5B	120.0	N1—C10—H10C	109.5
H5A—N5—H5B	120.0	H10A—C10—H10C	109.5
C7—C6—C5	122.39 (14)	H10B—C10—H10C	109.5
			
C8—N1—C2—N3	0.28 (18)	C10—N1—C8—C9	178.60 (13)
C10—N1—C2—N3	−178.55 (14)	C6—C7—C8—N1	177.97 (14)
N1—C2—N3—C9	−0.21 (18)	C6—C7—C8—C9	0.5 (2)
C9—C4—C5—N5	178.96 (12)	C2—N3—C9—C4	178.98 (14)
C9—C4—C5—C6	0.1 (2)	C2—N3—C9—C8	0.05 (17)
C4—C5—C6—C7	−0.5 (2)	C5—C4—C9—N3	−178.28 (14)
N5—C5—C6—C7	−179.37 (13)	C5—C4—C9—C8	0.6 (2)
C5—C6—C7—C8	0.2 (2)	N1—C8—C9—N3	0.11 (16)
C2—N1—C8—C7	−177.99 (16)	C7—C8—C9—N3	178.17 (13)
C10—N1—C8—C7	0.8 (3)	N1—C8—C9—C4	−178.94 (11)
C2—N1—C8—C9	−0.23 (15)	C7—C8—C9—C4	−0.9 (2)

### Hydrogen-bond geometry (Å, °)

**Table:**

*D*—H···*A*	*D*—H	H···*A*	*D*···*A*	*D*—H···*A*
N5—H5A···N3^i^	0.86	2.47	3.1447 (19)	136
C2—H2···N5^ii^	0.93	2.58	3.503 (2)	171

Symmetry codes: (i) −*x*+1/2, *y*−1/2, −*z*+1/2; (ii) *x*+1/2, −*y*+1/2, *z*+1/2.
